# Unusual Location of Neuroblastoma: A Report of Two Cases

**DOI:** 10.7759/cureus.70486

**Published:** 2024-09-30

**Authors:** Ahmed H Al Dhuhli, Ibtisam Al Shuaili, Khaloud H Abu Qasida

**Affiliations:** 1 Radiology, Royal Hospital, Muscat, OMN; 2 Radiology and Pediatric Radiology, Royal Hospital, Muscat, OMN; 3 Pathology and Laboratory Medicine, Royal Hospital, Muscat, OMN

**Keywords:** ataxia, chemotherapy, infantile neuroblastoma, pediatric hematology-oncology, urinary retention

## Abstract

Neuroblastoma is the most common extra-cranial solid tumor in children under the age of five years and is the second most prevalent malignancy in children after acute lymphoblastic leukemia. We are presenting two cases of neuroblastoma in children presented as intra-pelvic masses. The first patient presented with urinary retention while the second patient presented with ataxia. The initial imaging including ultrasound, CT scan, and MRI showed a large solid mass in the pelvis in both patients with extension to the spinal canal through the neural foramina at different levels. The histopathological results confirmed pelvic neuroblastomas. These cases highlight the rare locations of neuroblastoma in children and emphasize the variable presentations of this rare location. Knowledge of possible presentations of neuroblastoma aids early detection and expedites the proper management, especially in emergency settings.

## Introduction

Neuroblastoma is the most common extracranial solid tumor in children under the age of five years and is the second most prevalent malignancy in children after acute lymphoblastic leukemia [1]. 

The tumor arises from neural crest cells and can, therefore, occur anywhere along the sympathetic chain. It most commonly arises from the adrenal gland. Rarely, the tumor arises from the pelvis with reported incidence ranging from 2% to 7.5%. Pelvic tumors, along with cervical and thoracic tumors, are reported to have a better prognosis than abdominal tumors [2].

Patients with classic neuroblastoma usually present with symptoms of either local mass, sympathetic stimulations like opsomyoclonus, or symptoms of metastasis like pepper syndrome or blueberry muffin syndrome [3]. 

Patients with pelvic neuroblastoma can present with variable presentations including urinary retention, constipation, and ataxia [4]. The wide spectrum of presentations makes initial diagnosis difficult especially in emergency situations. Awareness of various possible locations of the tumor and its probable presentations improves early detection [1]. We are reporting two cases of pelvic neuroblastoma with different clinical presentations and histopathology findings.

## Case presentation

Case 1

A two-month-old infant with no previous medical background presented with a history of urinary retention and abdominal distention for three days. There was a remote history of long-standing constipation in the past six weeks with a frequency of passing stool every four to five days. There was no associated fever, cough, or vomiting.

On examination, the child was active and conscious with no signs of dehydration. Vital signs were within normal limits with normal temperature. A general examination showed no pain, respiratory distress, pallor, or jaundice. Chest examination was unremarkable. The abdomen was soft and distended with no masses felt. Urinary catheterization was done at the emergency room and approximately 160 mL of turbid-cooled urine passed with reliving of abdominal distention. Serum creatinine was increased to 133 umol/L, which was improved after catheterization to 37 umol/L. C-reactive protein was minimally elevated to 26.6 mg/L.

**Figure 1:**
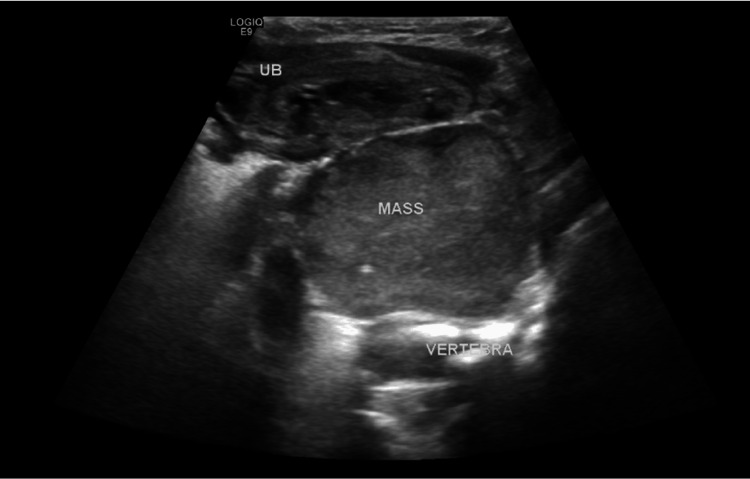
Mid-sagittal pelvic ultrasound scan with a large soft tissue mass (labeled) in the pelvis anterior to the vertebrae and posterior to the urinary bladder.

Initial imaging by ultrasound revealed a well-defined homogenous soft tissue mass posterior to the urinary bladder measuring 5.8x4.3x3.8 cm with few internal echogenic foci and mild vascularity in color Doppler study (Figure 1). This mass is seen completely intra-pelvic in the presacral region and causes a mass effect on adjacent structures pushing the rectum to the right side and the urinary bladder superiorly causing back pressure on the kidneys. The urinary bladder is collapsed with a thick trabeculated wall and internal debris.

**Figure 2:**
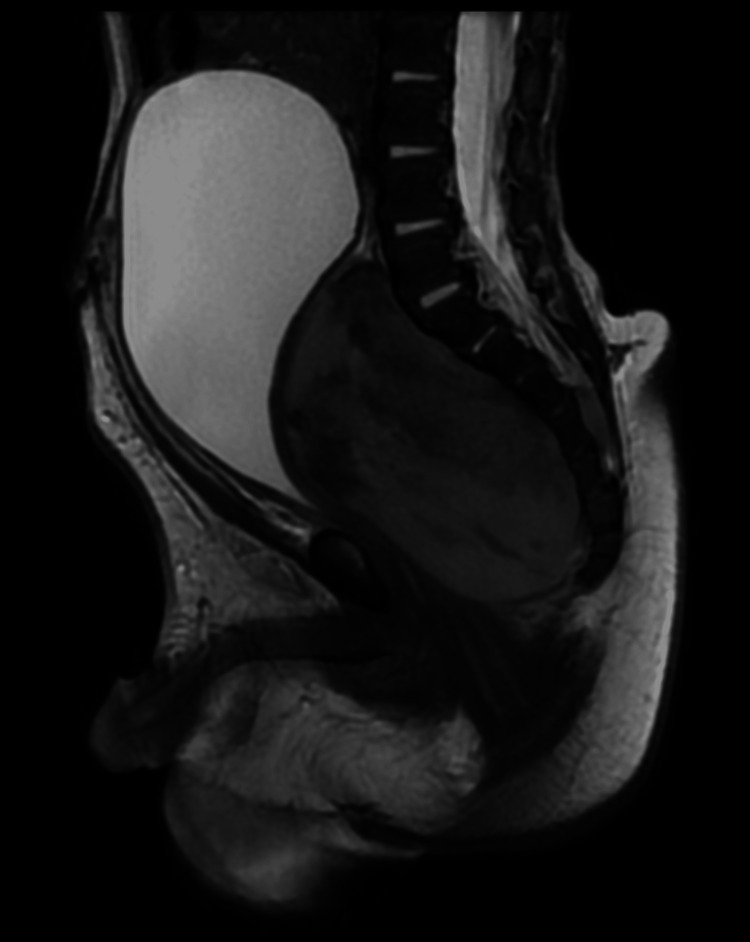
A sagittal image of the MRI scan revealed a large heterogeneous presacral mass extending to the spinal canal through the exiting neural foramina.

**Figure 3:**
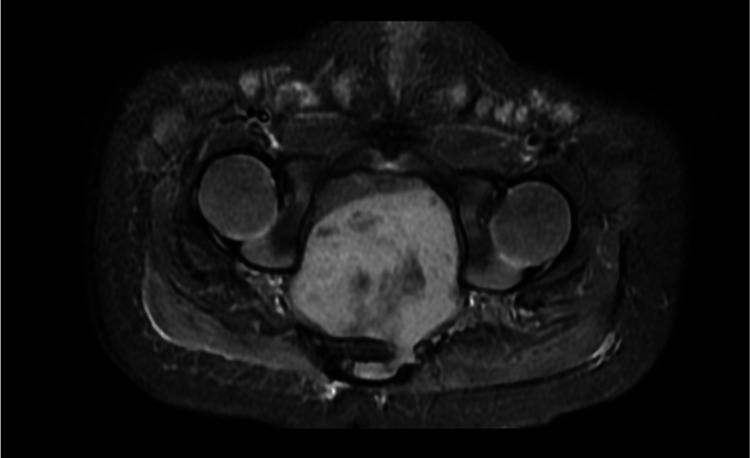
The axial image of the MRI scan revealed a large heterogeneous presacral mass extending to the spinal canal through the dilated neural exit foramina. No evidence of bone erosions.

MRI of the pelvis was recommended to further characterize the lesion. Pelvis MRI with IV contrast was done (Figures 2, 3), which showed a midline presacral soft tissue lesion extending craniocaudally from the level of L5 to coccyx measuring 6.1x3.2x4.1 cm in craniocaudal, anteroposterior, and transverse dimensions. The lesion is heterogeneously isointense to muscle in T1WI and heterogenous signal in T2WI. There was no significant drop of signal in STIR sequences signifying the absence of fat content. There are areas of internal hemorrhage within the lesion. Intense heterogenous enhancement is seen on post-contrast sequences. The lesion was extending posteriorly to the left neural foramina of at least three levels of the lower sacral bones. There was evidence of an intraspinal component at the lower sacrum. There was significant diffusion restriction. No evidence of bone marrow edema or sacrococcygeal involvement was seen. The initial impression was of a neurogenic pelvic tumor-like pelvic neuroblastoma; however, there was no characteristic enhancement pattern noted. No features of sacrococcygeal teratoma. Histopathological correlation was recommended.

Under ultrasound guidance, multiple core biopsies were taken from the lesion by the pediatric interventional radiologist, which confirmed the diagnosis of neuroblastoma.

Staging CT chest, abdomen, and pelvis was performed, which again showed the respective mass with mild heterogeneous enhancement and no definite calcifications or dense hemorrhage. There was no local or distant metastasis. The patient then underwent neoadjuvant therapy followed by surgical excision of the tumor.

**Figure 4:**
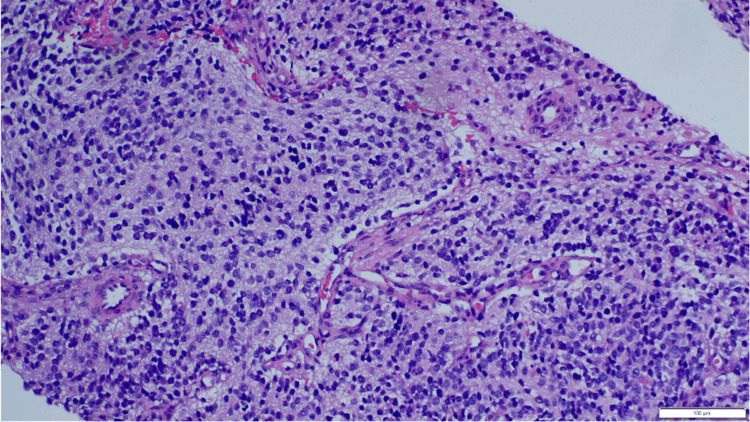
The biopsy shows sheets of small round blue cells in a neurofibrillary background (H&E, 20x).

**Figure 5:**
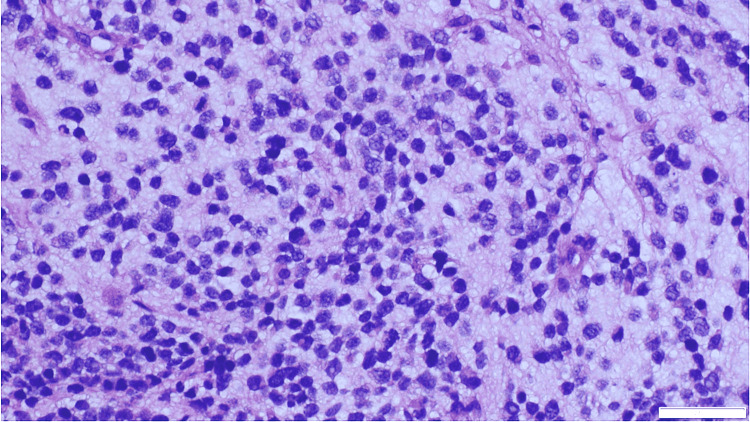
The neoplastic cells have a high nuclear-to-cytoplasmic ratio, hyperchromatic nuclei, and scant cytoplasm (H&E, 40x).

**Figure 6:**
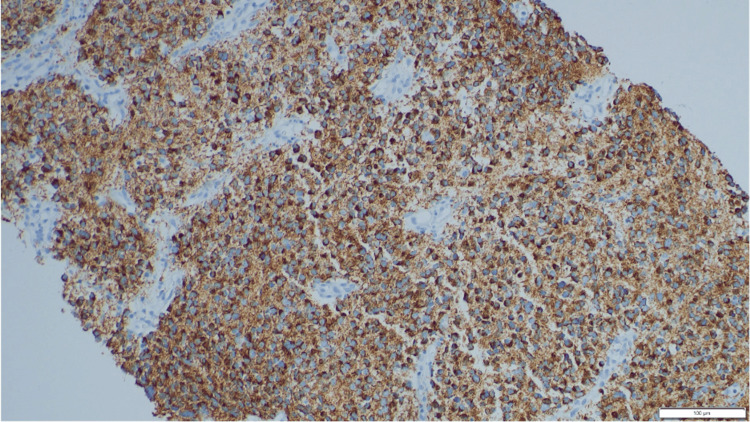
NB84 immunohistochemical stain is positive in the neoplastic cells (20x).

**Figure 7:**
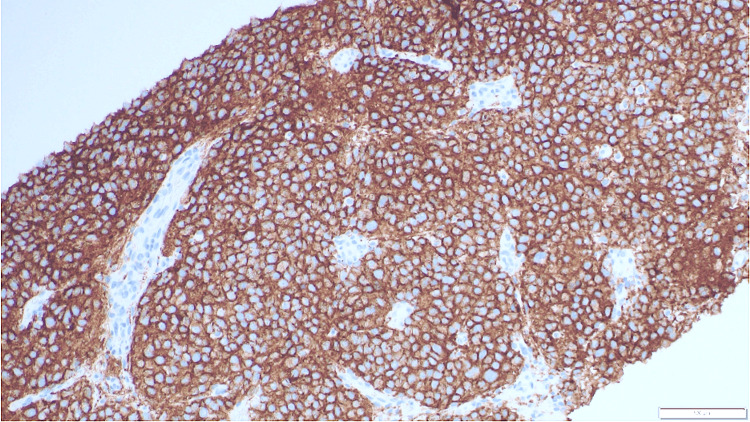
Synaptophysin immunohistochemical stain is positive in the neoplastic cells (20x).

Histopathological examination of the tumor revealed grossly a partly cut, opened, oval-shaped mass measuring 6.5x5x3 cm and weighing 63 g. The outer surface was smooth. Serial slicing revealed a grey solid surface with no hemorrhage or narcosis. Microscopic examination demonstrated a well-circumscribed tumor composed of differentiating neuroblasts surrounded by abundant neuropil, which is mixed with many maturing ganglion cells (Figures 4-7). No Schwannian stroma was identified. No necrosis was seen. The tumor was focally present at the resection margin. No lymph node was submitted for examination. The impression was poorly differentiated pelvic neuroblastoma with therapy-induced cyto-differentiation. 

Case 2

A two-year-and-six-month-old boy with no previous medical background presented to the emergency department with a two-day history of fever and a one-day history of ataxia. The fever was low grade and associated with mild coryza symptoms and good oral intake and activity. The child was noted to sway both sides while walking. The ataxia was noted to be static in course with no weakness, abnormal movements, or slurring of speech.

On examination, the child was sleeping comfortably and not in pain or distress. ﻿﻿The vital signs including the temperature were within normal limits. A neurological examination revealed an alert child with normal consciousness. ﻿﻿Ataxic gait was noted. The cranial nerve examination was unremarkable. Normal power of upper and lower limbs with no nystagmus or neck stiffness was noted.

Initial laboratory investigation showed normal blood counts, inflammatory markers, and renal function. Head CT was ordered and was reported unremarkable. The initial impression from the emergency department was acute ataxia with an element of post-viral cerebellitis.

**Figure 8:**
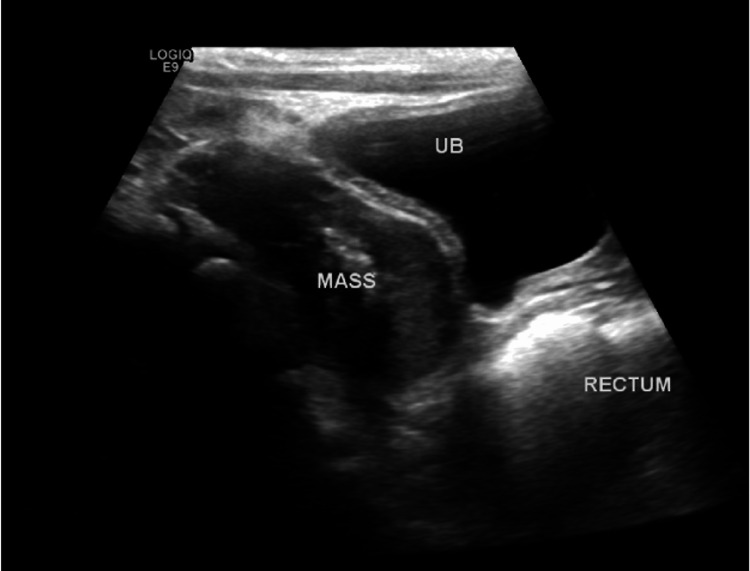
Greyscale ultrasound image demonstrating a large soft tissue mass (labeled) in the pelvis with internal vascularity and scattered calcifications.

Ultrasound was ordered by the treating team to exclude the remote possibility of neuroblastoma. The ultrasound scan (Figure 8) showed a well-defined, heterogeneously hypo-echoic, soft tissue mass lesion posterior to the urinary bladder and right side of the rectum displacing it to the left side. It was slightly lobulated with a smooth outline. It measured 4.9x3.4x2.9 cm in dimensions. Central calcifications of the lesion were noted. Mild vascularity was seen in color Doppler images. The differential diagnosis from the ultrasound included rhabdomyosarcoma or neuroblastoma, and further evaluation with cross-sectional imaging was advised.

**Figure 9:**
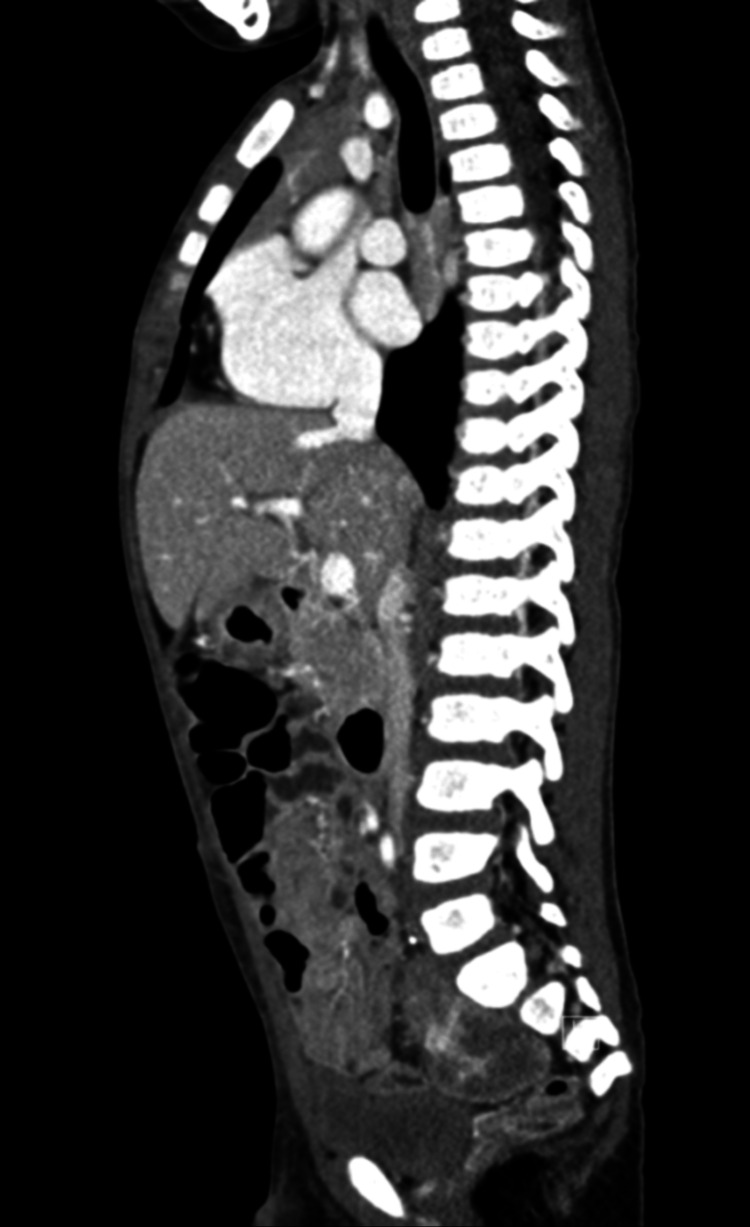
A sagittal image of the CT scan revealed a well-defined, hypodense, lobulated, heterogeneously enhancing, soft tissue mass in the pelvis.

Contrast-enhanced CT of the chest, abdomen, and pelvis was requested (Figure 9) and revealed well-defined, hypodense, lobulated, heterogeneously enhancing, soft tissue mass in the right side of the pelvis measuring about 4.5x3x3 cm with the tiny focus of calcification seen in the upper portion. It demonstrated close contact with the right sacral ala with possible extension to the L5/S1 neural foramen. There was no evidence of bony erosions or destruction. The mass is displacing the adjacent small bowel and rectum as well as the right internal iliac artery and branches laterally. No pelvic or abdominal lymph node enlargement is seen. No evidence of lung metastasis.

**Figure 10:**
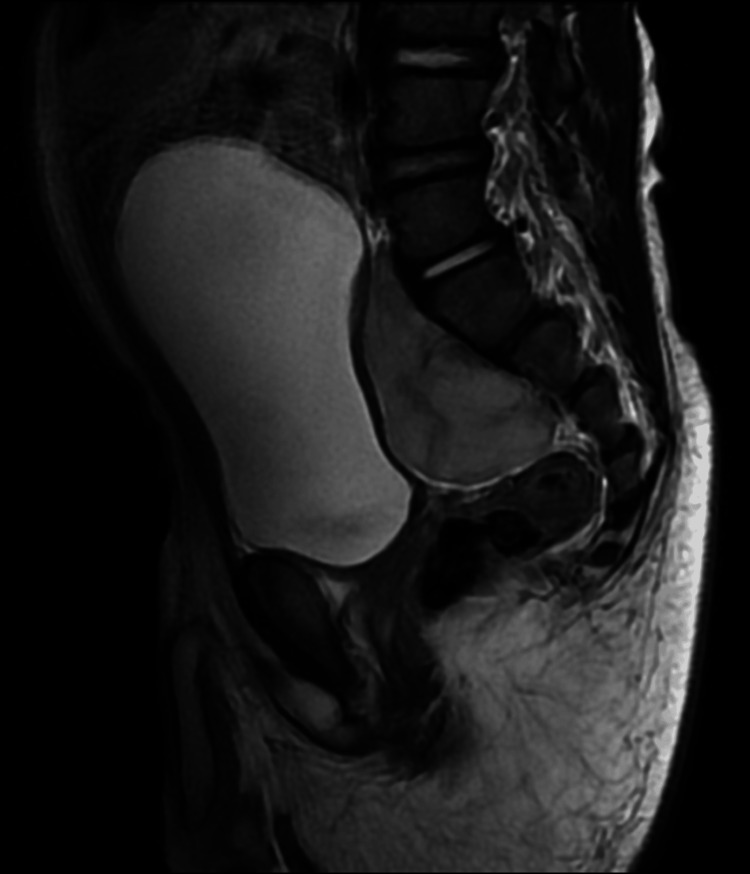
A sagittal image of MRI showed a solid mass on the right side of the pelvis posterior to the urinary bladder and was seen extending to the right sacral foramina.

**Figure 11:**
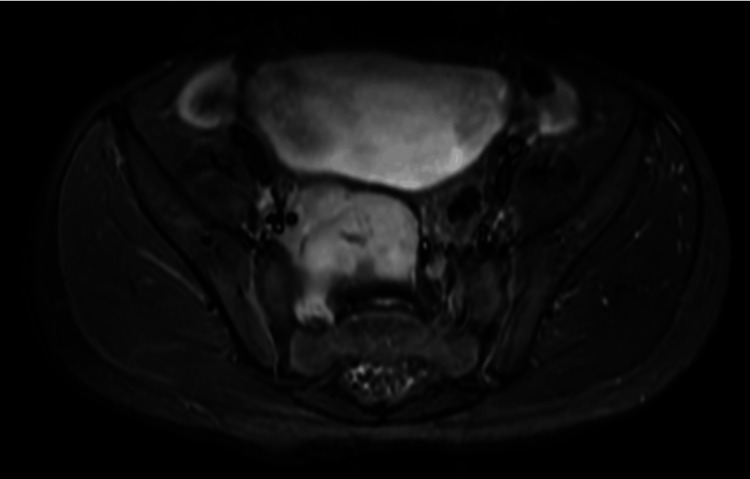
Axial image of MRI showed a solid mass on the right side of the pelvis posterior to the urinary bladder and seen extending to the right sacral foramina.

Contrast-enhanced pelvis MRI was performed (Figures 10, 11) and showed a solid mass on the right side of the pelvis measuring 4x3.4x5 cm. It was posteroinferior to the urinary bladder exerting mass effect upon the posterior wall; however, the clear fat plane is noted with no obvious signs of invasion. The rectum is displaced to the left side; however, its wall is separated from the mass with the clear fat plane. There is a clear extension of this mass through L5/S1 and S1/S2 neural foramina. The proximal segment of the right internal iliac artery and vein are encased and distally displaced by the mass; however, they appear patent with contrast enhancement. The mass demonstrated T1 isointense signal intensity and relatively high signal intensity on T2WI. Post-administration of contrast shows avid enhancement of the mass with evidence of diffusion restriction on diffusion-weighted sequences.

The patient underwent surgical resection of the tumor, and the tumor was sent for histopathological assessment. The histopathology laboratory received a well-circumscribed, encapsulated, light brown, firm mass weighing 18 g and measuring 5.2x3.1x2.6 cm. The outer surface was smooth and showed a few congested blood vessels. No breach in the capsule was noted. Serial slicing reveals a pale, vaguely lobular cut surface with a vague whorled pattern. No nodules were seen.

**Figure 12:**
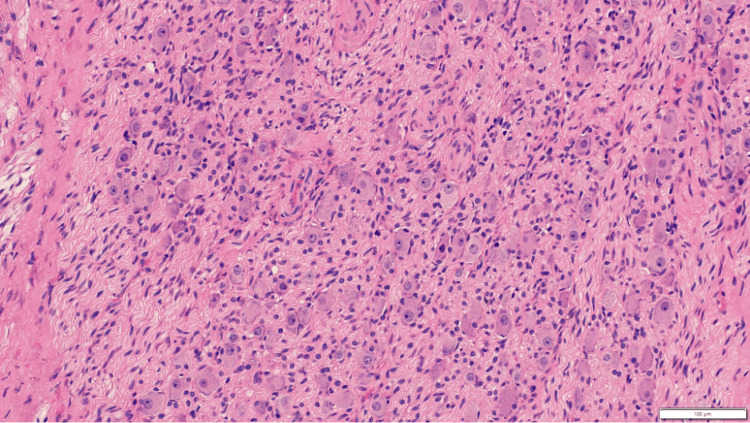
Maturing ganglion cells on a background of Schwannian stroma (H&E, 200x).

**Figure 13:**
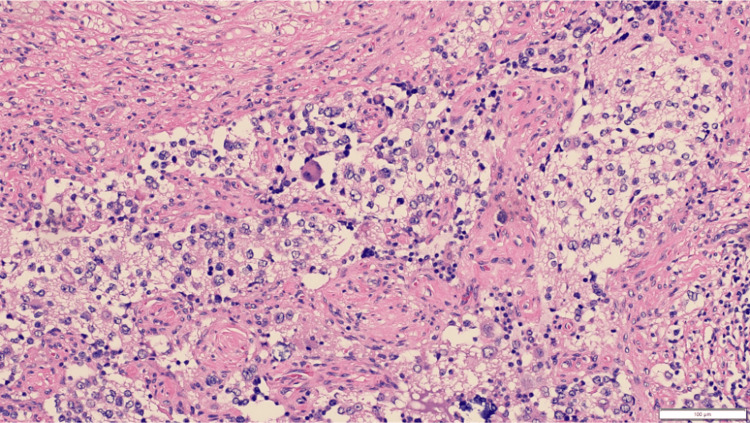
Maturing neuroblasts on a fibrillary background (neuropil) adjacent to an area with Schwannian stroma (top half) (H&E, 200x).

Microscopy sections showed a well-circumscribed neoplasm composed of many scattered, mature ganglion cells surrounded by abundant Schwannian-dominant stroma composed of spindle cells with comma-shaped, bland-appearing nuclei. Scattered lymphocytes with a few lymphoid aggregates were seen and small foci of calcifications were seen. A few blocks, in addition, showed a few small, microscopic nests of differentiating neuroblasts admixed with mature ganglion cells. No necrosis is seen. The mature neuroblastic component was seen within the adjacent adipose tissue and at the inked margin in a few areas. The histopathological impression was ganglioneuroblastoma, intermixed, of favorable histology (Figures 12, 13).

Post-operative I-131-MIBG and bone scan showed minimally MIBG avid residual presacral soft tissue thickening with no evidence of bone metastasis.

## Discussion

Neuroblastoma is the most common extracranial pediatric neoplasm. In the first year of life, neuroblastoma accounts for 50% of all tumors [3]. Neuroblastoma is associated with a favorable prognosis, with most patients considered to be at low or intermediate risk for recurrence of the disease [4]. However, 50% of patients are classified as having high-risk disease due to older age (>18 months) and metastatic disease or having locally advanced tumors with MYCN amplifications. The prognosis for high-risk patients is less favorable [5].

About half of the tumors arise from the adrenal gland; other sites include the organ of Zuckerkandl or along the paravertebral ganglia from the neck to the pelvis [6-9].

They have been associated with a number of disorders, such as Hirschsprung disease, fetal alcohol syndrome, DiGeorge syndrome, Von Recklinghausen disease, and Beckwith-Wiedemann syndrome.

CT scanning is the modality most commonly used to diagnose and stage neuroblastomas [10]. About 80-90% of neuroblastomas show stippled calcifications on CT.

MRI has some advantages over CT, such as no need for ionizing radiation; multiplanar imaging capabilities; and, often, the elimination of the need for IV contrast enhancement [11,12]. Obstetric ultrasonography can depict fetal neuroblastomas as early as 19 weeks' gestation. Most of the cases identified during obstetric ultrasonography are diagnosed during the third trimester (around 36 weeks).

Our patients have different locations of the neuroblastoma that are pelvic in origin, which itself is a rare presentation. Each patient had a different presentation ranging from ataxia to urinary retention. The first case has an elevated creatinine level, which may reflect urinary tract obstruction due to the neuroblastoma.

When an infant presents with reduced urine output and oral intake, an abdominal mass, protein in the urine, abnormal kidney function, and sudden urinary retention, pediatric urologists should suspect a pelvic neuroblastoma. A thorough history, physical examination, urinalysis, and imaging studies, such as ultrasound, CT scan, and MRI, are essential in cases of acute urinary retention. These evaluations may reveal a rare pelvic mass that necessitates surgical intervention, radiation, and/or chemotherapy.

## Conclusions

Neuroblastoma is a relatively common tumor in the pediatric age group; however, the variable possible locations and clinical presentations of the tumor make the initial diagnosis rather challenging. Intra-pelvic tumors are among the rare locations of neuroblastoma, which has peculiar presentations. Knowledge of those rare locations aids in the timely detection of the tumor and avoids the consequences of late diagnosis. Moreover, a detailed focus on history and clinical examination properly directs the investigations toward the correct diagnosis. Intra-pelvic sacrococcygeal teratoma is a great mimicker in some cases. 
